# Determining PTEN Functional Status by Network Component Deduced Transcription Factor Activities

**DOI:** 10.1371/journal.pone.0031053

**Published:** 2012-02-08

**Authors:** Linh M. Tran, Chun-Ju Chang, Seema Plaisier, Shumin Wu, Julie Dang, Paul S. Mischel, James C. Liao, Thomas G. Graeber, Hong Wu

**Affiliations:** 1 Department of Molecular and Medical Pharmacology, University of California Los Angeles, Los Angeles, California, United States of America; 2 Institute for Molecular Medicine, University of California Los Angeles, Los Angeles, California, United States of America; 3 Crump Institute for Molecular Imaging, University of California Los Angeles, Los Angeles, California, United States of America; 4 Department of Pathology and Laboratory Medicine, University of California Los Angeles, Los Angeles, California, United States of America; 5 Department of Chemical and Biomolecular Engineering, University of California Los Angeles, Los Angeles, California, United States of America; 6 Eli and Edythe Broad Center of Regenerative Medicine and Stem Cell Research, University of California Los Angeles, Los Angeles, California, United States of America; The University of Texas M.D Anderson Cancer Center, United States of America

## Abstract

PTEN-controlled PI3K-AKT-mTOR pathway represents one of the most deregulated signaling pathways in human cancers. With many small molecule inhibitors that target PI3K-AKT-mTOR pathway being exploited clinically, sensitive and reliable ways of stratifying patients according to their PTEN functional status and determining treatment outcomes are urgently needed. Heterogeneous loss of PTEN is commonly associated with human cancers and yet PTEN can also be regulated on epigenetic, transcriptional or post-translational levels, which makes the use of simple protein or gene expression-based analyses in determining PTEN status less accurate. In this study, we used network component analysis to identify 20 transcription factors (TFs) whose activities deduced from their target gene expressions were immediately altered upon the re-expression of PTEN in a PTEN-inducible system. Interestingly, PTEN controls the activities (TFA) rather than the expression levels of majority of these TFs and these PTEN-controlled TFAs are substantially altered in prostate cancer mouse models. Importantly, the activities of these TFs can be used to predict PTEN status in human prostate, breast and brain tumor samples with enhanced reliability when compared to straightforward IHC-based or expression-based analysis. Furthermore, our analysis indicates that unique sets of PTEN-controlled TFAs significantly contribute to specific tumor types. Together, our findings reveal that TFAs may be used as “signatures” for predicting PTEN functional status and elucidate the transcriptional architectures underlying human cancers caused by PTEN loss.

## Introduction

The *PTEN* (phosphatase and tensin homologue deleted on chromosome 10) tumor suppressor gene is mutated frequently in human cancers and cancer predisposition disorders [1], [2]. PTEN status not only pays a role in tumorigenesis, but also a crucial determinant for efficacy of cancer treatments. It has been shown that tumors with PTEN deficiency do not respond to Her2 inhibitor [3] or EGFR inhibitor [4] treatments whereas restored PTEN activity sensitized the tumor cells to these treatments. Accumulated evidence suggest that PTEN functional deficiency can result from different mechanisms, including *PTEN* genomic deletion, gene mutations, epigenetic silencing (e.g. silencing by DNA methylation or miRNAs), impaired membrane recruitment (e.g. loss of interaction with MAGI2) or decreased protein stability/activity mediated by various post-translational modification (e.g. phosphorylation, acetylation, oxidation, ubiquitination) [5], [6]. Therefore, it is rather difficult to determine PTEN functional status using simple gene expression or immunohistochemistry analysis.

Determination of PTEN functional status can be further complicated by the intricate signaling pathways that are regulated by PTEN. Through its lipid phosphatase activity, PTEN regulates PI3K-AKT-mTOR signaling that are involved in downstream transcription machineries, such as NF-κB, FOXO, and p53 [7], [8], [9], [10], [11]. PI3K-AKT signaling also engages other associated signaling networks and key factors responsible for cell size, cell motility, cell cycle, and cell death regulation [12]. Although regulation of PTEN-PI3K-AKT signaling cascade has been vigorously exploited, the multi-level controls of PTEN expression and activity and the complexity of feedback regulatory loops from PI3K downstream effectors to upstream receptor tyrosine kinase expression and activities have made determination of PTEN functional status and response of PTEN deficient tumors towards PI3K-AKT-mTOR pathway inhibition difficult. For example, inhibitors targeting PI3K, AKT and mTOR have been tested in multiple clinical trials [13]; however, it is recently reported that AKT inhibitors can induce the expression and phosphorylation of multiple upstream receptor tyrosine kinases (RTKs) whereas mTOR inhibitor rapamycin can activate AKT through a negative feedback mechanism [14]. Thus, the phospho-status of individual downstream signaling components, such as P-AKT and P-S6K, may not accurately represent PTEN status nor reflect the ultimate activation status of the PI3K/AKT signaling pathway.

To decipher functional status of PTEN and PTEN-controlled signaling network, we first analyzed the transcriptional targets which are immediately regulated by *PTEN* expression using global gene expression profiling in a *PTEN* inducible system. We further hypothesized that vast target gene expression changes may be controlled by a few key transcription factors (TFs), which can be a more sensitive and accurate “signature” for PTEN status. However, expression levels of these TFs will not always be sufficient to reflect their activity since the activity of a transcription factor (TFA) is controlled by various post-translational modifications as well as co-activator and co-repressor activities. Previous works by us and others have shown that TFA can be best inferred from the transcript levels of its direct target genes, rather than its mRNA level using Network Component Analysis (NCA) [15], [16], [17]. NCA is a model-based decomposition method to deduce transcription factor activity (TFA) and regulation control strength (CS) of TFs from target gene expression and information of TF - gene interactions. The information of TF - gene interaction, or network connectivity, is constructed by chromatin immunoprecipitation (ChIP) analysis [18], DamID methylation profiling [19] or extensive literature search [20].

**Figure 1:**
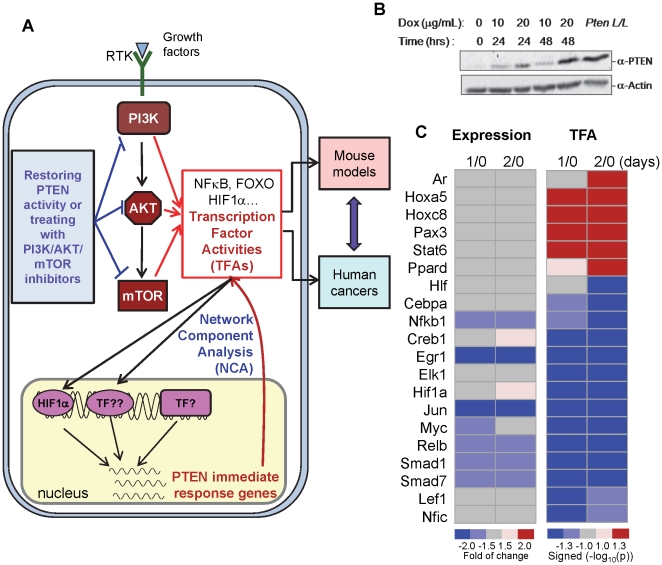
PTEN re-expression regulates transcription factor expression and activity in PTEN-inducible *Pten^Δloxp/Δloxp^* MEFs. (A) Schematic illustration of rationale and approach used in this study. To identify PTEN-controlled TFs, their activities (TFAs) in PTEN inducible system were first derived from expression of their target genes by NCA. The perturbed TFs were then examined further in mouse models and human cancers. (B) Immunoblot showing PTEN expression levels at 0, 1, and 2 days after treatment with the indicated concentration of Doxycycline in *Pten^Δloxp/Δloxp^* MEFs. Isogenic WT cells (*Pten^L/L^*) were used as a positive control. (C) Heatmaps showing the changes of expression and activity (TFA) of transcription factors in fold and log_10_ transformed p-value of the z-test, respectively, caused by PTEN re-expression for 1 day (1/0) or 2 days (2/0).

In this study, we identified 20 TFs whose activities are immediately altered upon the reexpression of *PTEN* (see Figure 1A for our overall strategy) in the *Pten* null mouse embryonic fibroblasts (MEFs). We found that the activities of these PTEN-controlled TFs are significantly altered in prostate cancer mouse models. Furthermore, the TFAs of these TFs show enhanced sensitivity and specificity when used to predict PTEN status in human prostate, breast and brain tumors, as compared to the gene expression-based analysis.

## Results

### Identification of transcription factors whose activities are perturbed by PTEN re-expression

To identify transcription factors whose expressions or activities are directly regulated by *PTEN* re-expression, an inducible system was generated in which *PTEN* expression can be controlled in a doxycycline-dependent manner in the *Pten* null *Pten^Δloxp/Δloxp^* cells [21]. PTEN protein level was significantly induced one day after 20 µg/ml doxycycline treatment and approached WT level on day 2 (Figure 1B; *Pten^L/L^* is a *Pten* WT line and isogenic to *Pten^Δloxp/Δloxp^*). Consistent to our previous study, re-expression of *PTEN* was able to suppress the gene expression of p90^MDM2^ isoform without significant change in p76^MDM2^ isoform as previously reported (Figure S1A) [21].

Next we analyzed global gene expression alterations immediate following *PTEN* re-expression by comparing gene expression levels 1 and 2 days after doxycycline (20 µg/mL) treatment with those before the treatment (Figure 1C; 1-0 and 2-0) and identified 352 genes whose expressions are changed by 2-fold within 2 days upon *PTEN* re-expression. Among these PTEN-induced genes, 136 (38.6%) are up regulated and 216 (61.4%) are down regulated by *PTEN* re-expression. Table S1 lists these genes along with their fold changes after inducing PTEN expression.

We reasoned that the PTEN-inducible genes must be regulated by the key transcription factors whose activities are controlled by PTEN. Therefore, we investigated PTEN immediately controlled TFs via network component analysis (NCA) in which the activities of TFs (TFAs) are deduced based on expression of their target genes. Different from conventional gene expression analysis, which focuses on statistically significant changes of individual genes, NCA deduces TFAs based on the concordant variations of all, rather than individual target genes, and that does not require the analyzed gene expressions to be statistically significant [15]. We recently developed a new NCA complementary algorithm [16] that allows analysis of mammalian datasets with limited number of data points for re-constructing a complicated network with multiple TFs. Specifically, this trimming algorithm removes the false positive interactions between TFs and their target genes, detected via high throughput ChIP-chip analysis, and enables the key target genes of the TFs of interest to be revealed [16].

We used NCA and the trimming algorithm to construct the transcription network, based on 70 known TFs and their controlled 782 target genes [20]. Heatmaps in Figure 1C shows that 20/70 TFs whose activities (TFAs) are immediately altered by *PTEN* re-expression (Figure 1C, right; p<0.05, 2-tail z-test). Interestingly, only Egr1 and c-Jun show >2-fold changes in their gene expression levels [22] and NF-κB, c-Myc, Relb, Smad1/7 show 1.5–2.0-fold of changes. These data imply that *PTEN* expression may influence the activities of these transcription factors without significantly changing their expression levels. Of note, several TFs, such as NF-κB and Hif1α, have been described as PTEN pathway-regulated TFs by previous reports [23], [24]. Unfortunately, Foxo, which is also known to be regulated by PTEN controlled AKT activity, is not included in this analysis since its target gene expression values are not available. We refer these 20 TFs as PTEN-controlled TFs whose activities depend on PTEN expression status.

### *PTEN* re-expression down-regulates transcription activities of c-MYC and LEF1

**Figure 2:**
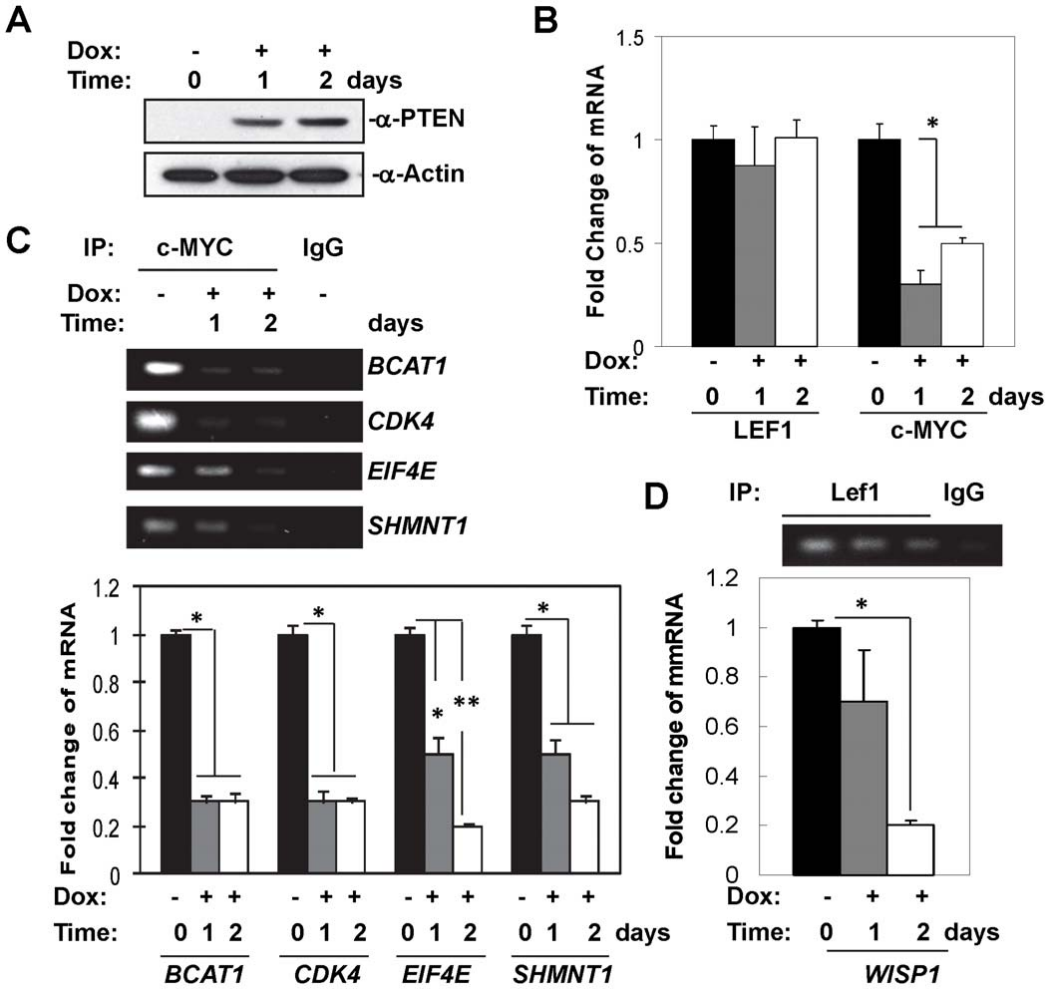
PTEN re-expression downregulates activities of c-MYC and LEF1 in PTEN-inducible PC3 cells. (A) Immunoblot showing PTEN expression levels under Doxycycline induction. (B) Bar graphs showing fold changes of c-MYC and LEF1 mRNA expression by qPCR analysis. (C and D) Bar graphs showing the target gene expression of c-MYC and LEF1 by qPCR analysis, respectively. *p<0.05 and **p<0.01.

To validate the PTEN-controlled TFs identified in *PTEN* inducible MEF cells, we employed another *PTEN*-inducible system, the *PTEN* null human prostate cancer cell line PC3 [6], [25]. By using human cell line, we are able to link PTEN-controlled TFs in mouse model to human cancers. Similar to our observation in the inducible-*Pten^Δloxp/Δloxp^* MEFs, PTEN protein expression is significantly induced after 1 and 2 days of doxycycline treatment in the inducible-PTEN PC3 cells (Figure 2A). Using qPCR analysis, we found that the expression levels of *c-MYC* and *LEF1* after PTEN re-expression in PC3 cells also echoed those from the MEF-derived data: *c-MYC* gene expression is down-regulated (p<0.05) while *LEF1* expression level remained constant (Figure 1C and 2B). We also measured the total protein level of c-MYC and phosphorylated to total protein ratios of STAT6 and c-JUN and found no substantial changes in c-MYC and STAT6 after PTEN re-expression (Figure S1B). In contrast, the ratio of phospho-c-JUN to total c-JUN is reduced by more than 2-fold after *PTEN* re-expression, consistent with our previous study [26].

The MEF system results predict that transcription activity of c-MYC would be down regulated by *PTEN* re-expression in the PC3 system. To validate this, we examined the association between c-MYC and the promoters of its target genes using chromatin immunoprecipitation (ChIP) analysis. c-MYC target genes *BCAT1*, *CDK4*, *EIF4E*, and *SHMNT1* were selected because (1) their proximal promoter regions contain c-MYC consensus binding sequence (CACGTG), which is conserved between human and mouse; and (2) the control strengths by c-MYC were highly significant based on NCA analysis (Figure 1C). We found that *PTEN* re-expression significantly decreases the association of c-MYC to the promoter of its target genes (Figure 2C top panel), and that in turn reduces expressions of these c-MYC target genes (Figure 2C bottom bar graph). Since *PTEN* re-expression does not change the total c-MYC protein level, PTEN must modulate c-MYC transcription factor activity by either regulating the half-life of c-MYC or the activities of its binding partners. Similarly, *PTEN* re-expression also decreases the association of LEF1 to the promoter of its target gene *WISP1*, and that leads to concurrent reduction of its gene expression (Figure 2D). These results suggest that transcription activities of both c-MYC and LEF1 are perturbed by *PTEN* expression. Furthermore, our data show that in addition to transcriptional activity, *PTEN* induction also reduces *c*-*MYC* gene expression, suggesting PTEN-mediated suppression of c-MYC activity might be partially contributed by its decreased expression. However, PTEN appears to impact solely on LEF1 activity without altering mRNA expression of the transcription factor itself.

### The activities of PTEN-controlled TFs are substantially altered in murine prostate cancer models *in vivo*

**Figure 3:**
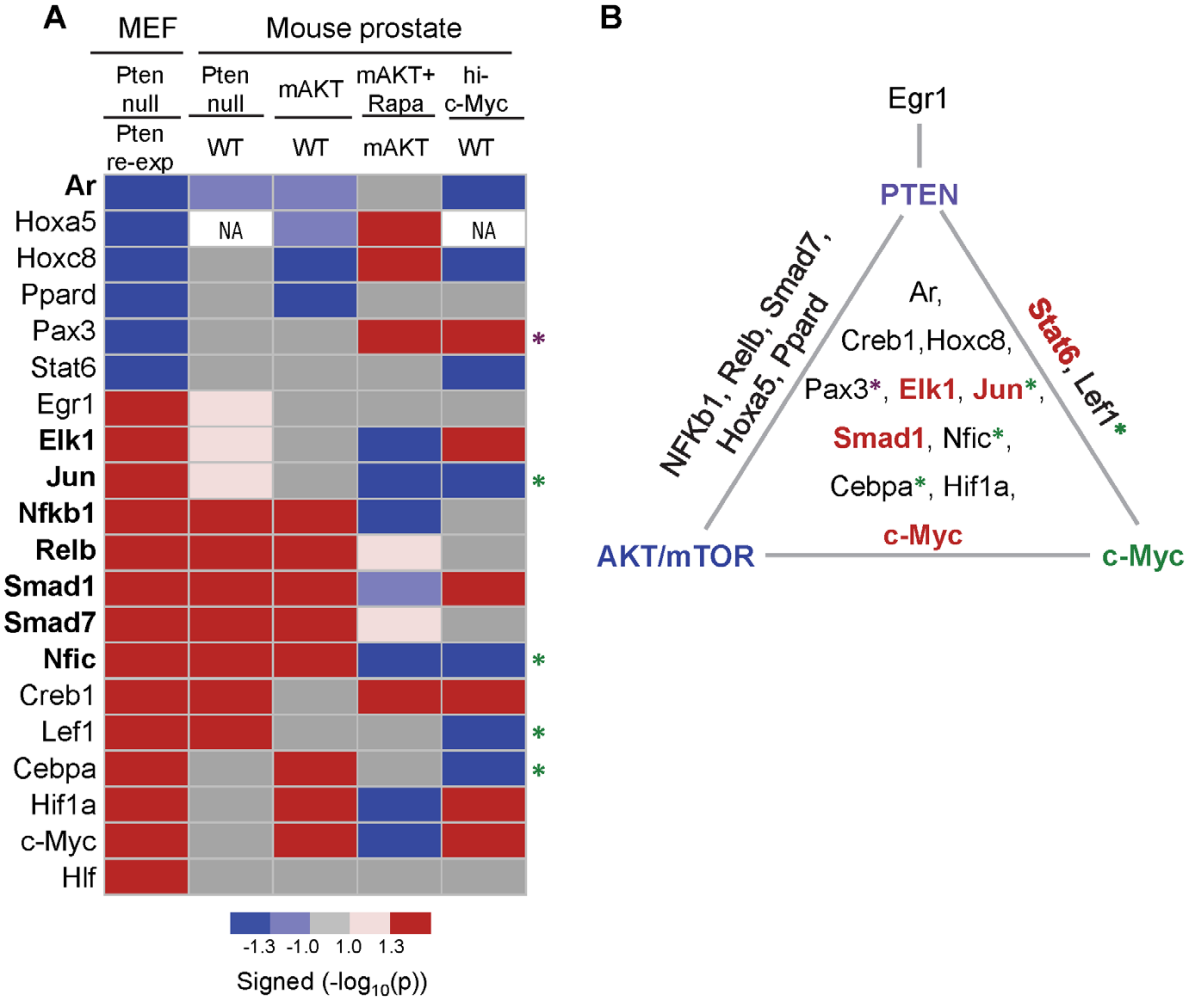
PTEN-regulated TFAs are significantly increased in murine prostate cancer models *in vivo*. (A) Heatmap showing changes of PTEN-regulated TFAs in PTEN inducible MEFs (PTEN null compared to PTEN re-expression or PTEN WT) and murine prostate cancer models (compared to WT control mice; Rapa: Rapamycin treatment). TFAs regulated by PTEN/AKT/mTOR pathway are marked in bold. TFAs exhibit discordant regulation between c-Myc and the PTEN/AKT/mTOR pathway are marked by *. The purple and green asterisks indicate Myc-activating and suppressing TFs respectively. (B) Triangle diagram summarizing the TFAs regulated by PTEN, AKT/mTOR and/or c-MYC.

PTEN plays a critical role in human and murine prostate tumorigenesis [1], [27], [28]. To determine whether TFAs deducted from *PTEN*-inducible MEF reflect PTEN functional status *in vivo*, we examined the TFAs, based on the gene expression datasets we have in hand, of three well-established murine prostate cancer models, i.e. the *Pten* null [29], the *mAKT1* [24], and the *hi-c-Myc* [30] models (Figure 3A), and then categorized the TFs into subgroups according to alterations of their activities in these mouse models (Figure 3B). Figure S2 illustrates the TFA profiles in all three murine models. As expected, the total numbers of perturbed TFs in murine cancer models is higher than that in PTEN inducible system. Among the 20 PTEN-controlled TFs identified in the *Pten* MEF system, all except Hlf are significantly altered in at least one of the models. Egr1 has its activity altered solely in the *Pten* null murine model. Eight TFAs (marked in bold in Figure 3A) exhibit concordant alterations when the PTEN downstream AKT/mTOR pathway is manipulated genetically or pharmacologically (Rapamycin treated *mAKT1* model). Interestingly, *hi-c-Myc* shares 13 TFAs changes with *Pten* null or *Pten* null and *mAKT1* models, of which seven are concordantly regulated, including Ar, Creb1, Hoxc8, Elk1, Smad1, Hif1α, Stat6 and c-Myc (Figure 3B). Since c-Myc transcription level and activity is regulated by PTEN, these seven TFAs may reflect the epistatic component of PTEN/AKT/mTOR and c-Myc. The remaining 5 TFAs (* in Figure 3A and B) are discordantly regulated by c-Myc and the PTEN/AKT/mTOR pathway, either due to non-overlapping function and/or additional alterations accumulated in vivo during tumor initiation and progression of these in vivo models. In brief, the activities of the majority of PTEN-controlled TFs are altered in the murine prostate cancer models in vivo, and the variations among the models might be explained by different genetic background and stage of tumor development.

### Signature of PTEN-controlled TFs correlates with PTEN status in human cancers

We hypothesized that the TFAs controlled by PTEN expression should reflect PTEN functional status in human cancers. To test the hypothesis, we examined if tumor subgroups determined by TFA-based unsupervised clustering are enriched for tumors with distinct PTEN status. For this, we focused on prostate, breast and brain tumors because 1) PTEN deficiency frequently occurs in these cancers [27], [31], [32], and 2) large gene expression datasets are publically available [32], [33], [34]. Out of 19 PTEN-controlled TFs (AR is removed from our analysis to prevent bias toward prostate), we were able to derive 16, 15 and 16 TFAs from human prostate, breast and brain tumor datasets, respectively, based on the availability of their target gene expression values in the datasets. For each cancer type, patient samples were first classified by unsupervised clustering based on the signature TFAs then annotated with their associated pathological grades or PTEN status, determined by either CGH analysis for PTEN copy number (CN) alterations in prostate cancers [35] or immunohistochemistry (IHC) and mRNA array for PTEN expression in breast cancers [32]. For brain tumors, PTEN status was first predicted by TFAs-based unsupervised clustering, and then confirmed by us in a majority of the samples by IHC analysis.

**Figure 4:**
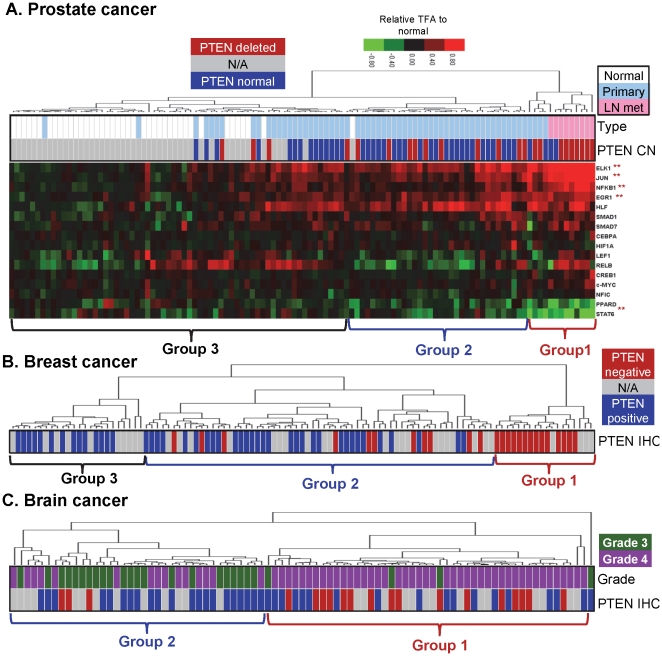
PTEN-controlled TFAs predict PTEN status in human cancers. Unsupervised clustering analysis, based on PTEN-controlled TFAs, was used to classify human tumor samples. (A) In prostate cancer, group 1 is largely composed by samples with PTEN copy number changes (CN, red) and lymph node metastases (LN met, pink); Group 2 are primary cancer samples (light blue) with normal PTEN karyotype (blue) that are separated from most of normal prostate tissues (white). TFAs that are significantly altered between group 1 and group 3 are mark by **, p<0.001. The heatmap was plotted based on relative changes to the respective average TFAs of normal samples. (B) In breast cancer, group 1 is mostly comprised of samples with PTEN-negative status (red) identified by immunohistochemistry (IHC). The majority of the samples in group 3 have positive PTEN status (blue), while group 2 includes both positive and negative PTEN samples. (C) In brain tumors, most samples in group 1 are associated with PTEN negative status (red). The PTEN negative subgroup is also correlated with higher tumor grade (green for grade 3 and purple for 4, respectively).

As shown in Figure 4A, PTEN-controlled TFAs separate 112 prostate samples into three distinct groups. Group 1 contains mostly lymph node metastatic samples in which 69% (9/13) have deleted PTEN based on CN; Group 2 has mostly primary cancer samples in which 31% (11/35) have PTEN CN changes, whereas Group 3 consists of a majority of normal prostate cancer samples with only 4.7% (3/64) PTEN CN changes. Of note, the overall rates of PTEN CN alterations found in this cohort fit well with the results from integrated genomic profiling of a larger group of human prostate cancer samples [27]. The heatmap of clustered TFA levels shows that the activities of EGR1, ELK1, JUN, and NF-κB1 are significantly (**, t-test p<0.001) higher in PTEN negative samples while STAT6 activity is lower in PTEN negative samples, as compared to those in the PTEN positive samples (Figure 4A).

PTEN-controlled TFA patterns can also be used as signatures to separate PTEN negative from PTEN positive breast cancers (Group 1 vs. Group 3 respectively in Figure 4B and S3A). The same TFA-based analysis on Netherland Cancer Institute (NKI) published breast cancer dataset that does not have associated PTEN status [36], also classifies breast cancer in three subgroups, and the Group 1, predicted to be PTEN negative, is associated with poor differentiated, ER-negative basal-like phenotype [37] (Figure S3B), which is consistent with our recent publication [38].

**Figure 5:**
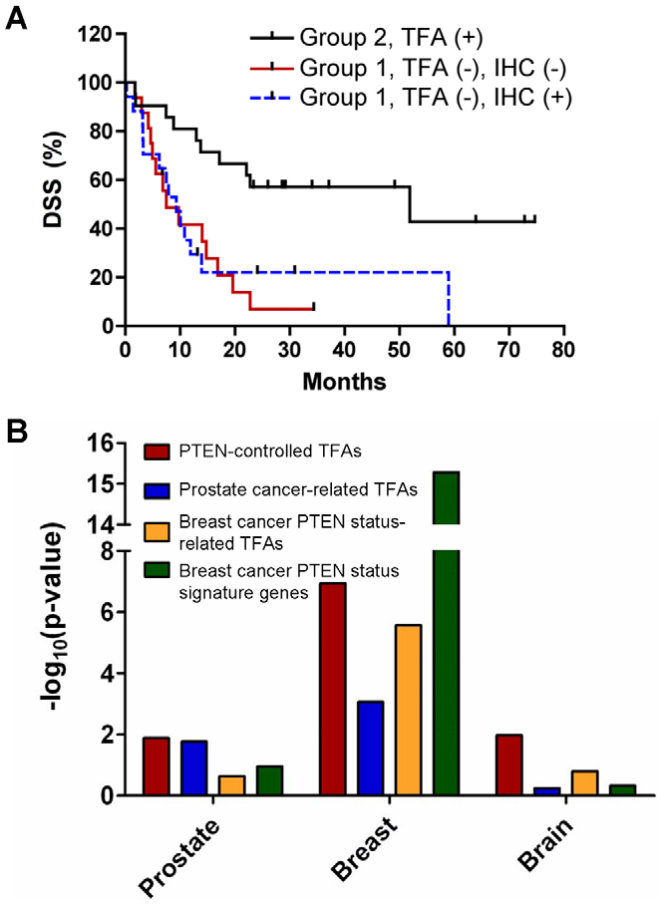
Enhanced robustness of TFA-based signatures in predicting PTEN status in human cancer. (A) The Kaplan-Meier survival curves of patients with brain tumors stratified according to PTEN-controlled TFA and IHC analyses. (B) Log_10_ transformed p-values of the χ^2^ test evaluating the association of PTEN status with the hierarchical clustering-determined groups of human tumors. Clustering results are based on PTEN-controlled TFAs (red; Figure 3), prostate cancer-related TFAs (blue), TFA-based (gold) and gene expression-based (green) signatures derived from PTEN IHC data in breast tumors. When three major clusters are observed in prostate and breast cancers, the χ^2^ tests are performed to associate different PTEN status in group 1 and groups 2 plus 3.

Similarly, PTEN-controlled TFAs can separate grade 4 glioblastomas from grade 3 gliomas (Group 1 vs. Group 2 in Figure 4C and S3C). As expected, patients within the PTEN TFA-positive group (Group 2) have mostly grade 3 tumors and longer disease specific survival (DSS) while patients within the PTEN TFA-negative group (Group 1) have more aggressive tumors and shorter DSS. We then compared DSS of patients within Group 1 whose PTEN status were determined by IHC analysis (PTEN IHC positive = blue bar, 17 patients; PTEN IHC negative = red bar, 16 patients) and found that PTEN IHC-positive patients have nearly identical DSS as PTEN IHC-negative patients (log-rank test p = 0.8, Figure 5A). Thus, PTEN IHC status does not significantly segregate patients in terms of the functional outcome of DSS, while PTEN TFA status does. This analysis illustrates the advantage of using TFA-based signatures in evaluating PTEN functional status over traditional IHC-based analysis. Together, these results reveal the power of our approach in predicting PTEN status and its pathological association in human cancers in general.

### Predicting PTEN status using murine and human cancer deduced TFA signatures

In addition to the PTEN-controlled TFAs derived from the inducible *Pten^Δloxp/Δloxp^* MEFs, we identified another 19 TFs whose activities are significantly perturbed in the *Pten* null prostate cancer mouse model (Figure S2). These TFAs are not changed by transient *PTEN* re-expression in the MEF cell line. Interestingly, 16 out of these 19 TFAs are concordantly regulated in the *mAKT1* and *hi-c-Myc* models and are also altered upon rapamycin treatment of the *mAKT1* model. Since these TFAs are not subject to the control of PTEN re-expression and may reflect the common pathological changes associated with these prostate cancer models, we coined them “prostate cancer-related TFAs”. We tested the power of both the PTEN-controlled TFAs that were defined in the Pten inducible MEF system, and the prostate cancer-related TFAs in predicting PTEN status in human cancers. The prostate cancer-related TFA signatures can be used to classify PTEN status in human prostate cancers to a similar accuracy as the PTEN-controlled TFAs, but they had less cross-tissue predictive power as they failed to reach significance levels when applied to breast and brain tumors (Figure 5B, blue bars).

Gene expression signatures, extracted from dysregulated genes in PTEN deficient cancer samples, have been used to predict PTEN status in human breast cancers [32]. We therefore, compared gene expression signature-based predictions to inferred TFA signature-based predictions of PTEN status in human cancer samples. Applying our trimming NCA algorithm to the breast data set annotated with PTEN IHC status, we identified 15 TFs whose inferred TFAs were significantly altered (t-test p<0.0001) (Figure S4). Among these six, HLF, JUN, c-MYC, EGR1, SMAD1, and HIF1A, were also identified as PTEN-controlled TFs (Figure 1C), and four, ESR2, MYB, RELA, and USF1, as prostate cancer-related TFs (Figure S2). 246 signature genes predictive of PTEN status had been previously reported in the breast cancer dataset [32]. Among these, 103 and 123 genes, respectively, had their expression values measured in the prostate and brain cancer datasets. As shown in Figure 5B, although both gene expression-based (green bars) and TFA-based (gold bars) breast PTEN IHC-based signatures can be used to predict PTEN status by unsupervised clustering approach in human breast cancers, they failed to do so for prostate cancers and brain tumors. These results demonstrate that the transcriptional network-inferred PTEN-controlled TFAs are generally more reliable than expression-based gene sets in representing PTEN functional status.

### Specific sets of PTEN-controlled TFAs preferentially contribute to different tumor development

**Figure 6:**
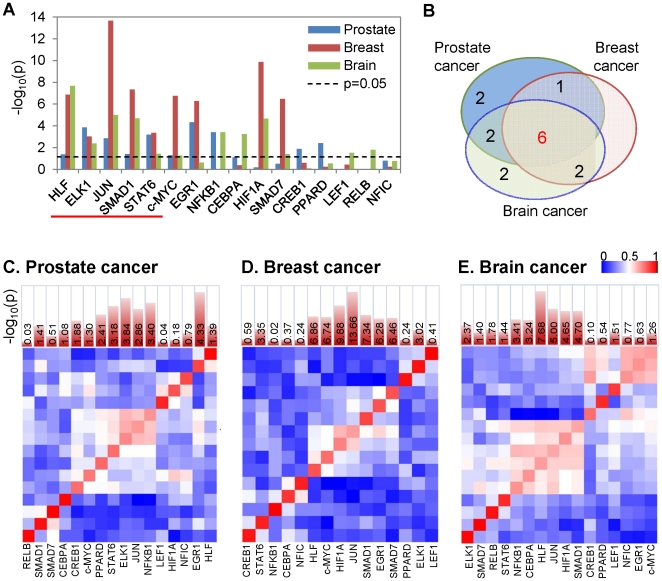
Subsets of PTEN-controlled TFAs preferentially function in specific types of tumors. (A) t-test p-values comparing TFAs of the subgroups based on PTEN status and clustering results as shown in Figure 5 in three tumor types. In the t-tests performed on of prostate and breast cancers, PTEN positive samples in group 3 were used as the PTEN positive functional status, the PTEN negative samples in group 1 as the PTEN negative functional status. Similarly, in brain tumor PTEN IHC positive samples in group 2 and PTEN IHC negative samples in group 1 were selected for representing PTEN positive and negative functional status respectively. The red line highlighted the 6 TFAs significantly (p<0.05) altered between tumor subgroups in three tumor types. (B) Venn diagram summarizing the overlap of the TFAs that contribute to the discrimination of tumor subgroups with different PTEN status in different tumor types. (C–D) Heatmap of the absolute Pearson correlation coefficients between NCA-inferred TF activity profiles across the tumor samples from prostate (C), breast (D) and brain (E) cancers, indicating groups of co-active transcription factors may function together.

Given that the PTEN-controlled TFA signatures are associated with PTEN status in prostate, breast and brain tumors, we next asked if a particular subset of the transcription factors play more important role in each individual tumor type. To this end, we first compared each TFA between PTEN positive and negative samples, identified by both IHC/CN and TFA-based analysis. Figure 6A shows the log_10_ transformed p-value of the t-test of such comparisons in each tumor type. The first six TFAs, i.e., HLF, ELK1, JUN, SMAD1, STAT6 and c-MYC, are significantly (p<0.05) altered in the PTEN negative group in all three cancers. STAT6 TFA is decreased while the others TFA are increased in the PTEN negative group. The degree of overlap of the tumor type-specific PTEN-controlled TFAs is summarized by a Venn diagram in Figure 6B.

We further investigated the possible interactions among the TFAs by examining pair wise correlation coefficients of inferred TFAs across patient tumors in each tissue type. The absolute correlation coefficients between the pairs are illustrated in Figure 6C–E for human prostate, breast and brain tumors, respectively. In general, the TFAs that more significantly track with PTEN status are highly correlated with each other, and assemble together into clusters that may reflect common upstream signaling-based activation mechanisms. Notably, in each tumor type, more than one TFA clusters are formed, suggesting that PTEN regulates TFAs through more than a single signaling pathway. The overlapping and specific TFA signatures in different types of human cancers provide a transcription factor-based guide to the mechanisms of cancer development caused by PTEN loss, and offer TFA-based rationales for designing new therapeutic regimen for treating PTEN null cancers and also monitoring PI3K pathway targeted treatment responses.

## Discussion

In this study we used NCA and its complementary trimming algorithm to reveal 20 TFs that immediately respond to the expression of PTEN in a PTEN inducible system. We found that the PTEN immediate responsive gene-based TFA signatures are more accurate and sensitive than either cancer-based TFA or gene expression-based analyses in predicting PTEN functional status in human cancers. These TFA-based signatures, therefore, provide readout of transcription factor activity even if their mRNA levels do not change, and help to overcome the complexity of multifactorial post-transcriptional PTEN signaling pathway regulations. Since mRNA profiles are currently measureable in clinical settings, our TFA-based signatures provide new rationales for stratifying patients according to their PTEN functional status and for monitoring treatment outcome in PI3K-targeted therapies.

Our study testifies to the power of combining traditional genetic and biochemical approaches with mathematic algorithms in deciphering complicated transcription regulatory networks. NCA complements other classical bioinformatics methods, such as Principal Component Analysis (PCA) and Independent Component Analysis (ICA). In contrast to PCA and ICA, NCA utilizes biochemical constraints, i.e., the relationship between transcription factors and their regulated genes, rather than statistical or mathematical constraints in data deconvolution. This means if target genes of a certain TF concordantly altered their expressions, even though at the subtle levels upon stimulation, its NCA-derived TFA will show significantly changes. Besides, NCA also detangles effects of multiple TFs regulating a same gene. TFA profiles are more robust and reliable in representing the real activities than the expression of a TF specific target gene which may not be expressed in all tissues. This explains why TFA-based signatures are more reliable than gene-based signatures in predicting PTEN status in different tumor types. NCA has been used to reveal biological relevant network structure and regulatory dynamics in bacteria [17], [39], [40], [41], [42], *Saccaramyces cerevisiae* [41], [43] and mouse [44]. In our study, the PTEN-controlled TFA signatures deduced by our NCA analysis have been validated experimentally and bioinformatically across in vitro cell lines, in vivo animal models and in patient samples.

A shortcoming of NCA is that it depends on the information of TF and target gene relationship. For instance, although FOXO activity is known to be regulated by PTEN controlled PI3K/AKT pathway, FOXO TFA cannot be derived in PTEN inducible system because its target gene expression values are not available in the database we used. Nevertheless, the results obtained from our analyses are quite robust.

Although PTEN is not a TF, it can regulate TFAs through either phosphatase-dependent or -independent mechanisms. In the prostate cancer mouse models, the majority of TFs perturbed in the *Pten* null mouse exhibit concordant alterations in their activities when the PTEN downstream AKT/mTOR pathway is manipulated genetically or pharmaceutically by the mTOR inhibitor rapamycin. This result implies that PTEN controls TFAs through its phosphatase activity by regulating the PI3K/AKT/mTOR pathway, which is known for regulating activities of several TFs including NF-κB [23], p53 [10], FOXO [9] and CREB [45]. These TFs may also serve as co-activators or repressors for other TFs. Interestingly, we found c-MYC level and activity is directly regulated by PTEN expression and a significant overlap between *hi-c-Myc* and *Pten* null or *mAKT1* prostate cancer models in their associated prostate cancer-associated TFAs. It is worthy to note that c-MYC target genes that are perturbed by *PTEN* expression involve the regulation of cell growth, cell metabolism, and protein synthesis [46], [47], [48], [49], suggesting that the c-MYC-regulated target genes may potentially play important roles in the development of PTEN-deficient cancers. PTEN can also function independent of its phosphatase activity to regulate cell migration [50], p53 [10] and APC-CDH2 [51]. It is conceivable that PTEN regulates a subset of TFAs, such as EGR1, LEF1, HLF, and STAT6 (Figure 3), through its phosphatase-independent functional domains [6]. Whether PTEN needs to be located in the nuclear to perform such a function requires further investigation.

Our study reveals common and cancer tissue type-specific regulation of TFAs by the PTEN tumor suppressor. The six common PTEN-controlled TFAs including c-MYC most likely play an essential role in tumor development caused by PTEN loss and their activity may serve as surrogate markers for determining PTEN functional status and measuring response to targeted therapy. Other tissue-type-specific regulated TFAs may help us understand PTEN's tissue-specific function. The PTEN-controlled TFAs deduced by NCA, therefore, will aid in stratifying cancer patients according to PTEN functional status and in deciphering the complicated transcription regulatory networks controlled by the PTEN tumor suppressor. Although targeting transcription factors with small molecules remains challenging, recent works by Bradner and colleagues on selective inhibitions of BET bromodomains [52] and NOTCH transcription factor complex [53] provide promising direction of this approach. Since efficient targeting the intermediate effectors of the PTEN controlled signaling pathway is difficult because of the feedback loops and cross-talks among intracellular signaling pathways, our study may guide the future efforts in targeting PTEN deficient cancers through disrupting its regulated transcriptional architectures.

## Materials and Methods

### Cell culture and transfection

*PTEN*-inducible mouse Pten^Δloxp/Δloxp^ MEF cells [21] or PC3 [6], [54] cells were cultured in DMEM or RPMI medium, respectively, supplemented with 10% tetracycline-free fetal calf serum (Invitrogen), 100 U/ml penicillin and streptomycin (Gibco).

### Real-time PCR

Total RNAs were extracted using RNeasy Mini kit (Qiagen). RNAs were reverse-transcribed by oligo(dT) primer using Superscript RT-PCR kit (Invitrogen), according to the manufacturer instructions. PCR reaction was performed under the following conditions: 94°C for 3 min; 94°C 30 Sec; 58°C for 30Sec; 72°C for 30 Sec for 40 cycles; and 72°C for 10 min, using iQ SYBER Green Supermix Kit from Bio-Rad. Results were analyzed by the relative quantification method and expressed as relative RNA levels (ΔCT, difference of cycling threshold). ΔCT values represent CT [gene]-CT [GAPDH], thus higher values indicate relatively lower expression levels. Primer sequences used for real-time PCR were retrieved from PrimerBank website.

### Chromatin immunoprecipitation (ChIP) assay

Chromatin immunoprecipitations were modified from the EZ- ChIP (Upstate) protocol using antibodies: anti-c-Myc (A14, Santa Cruz) anti-LEF1 (C-19, Santa Cruz). The percentage of the bound DNA was quantified against the original DNA input using real time PCR analysis (Biorad). Primer sequences used for ChIP are as follows:

BCAT-5′: AATCCGCTAGGTCGCGAGT; BCAT-3′: AGCAAGACCTGGGGCAGT

CDK4-5′: TTACACTCTTCGCCCTCCTC; CDK4-3′: ATGTGACCAGCTGCCAAAG

EIF4E-5′: CAGGGCCAAACGGACATA; EIF4E-3′: CAATACTCACCGGTTCGACA

SHMNT1-5′: GCAGAGTGCACCTTCCTGA;

SHMNT1-3′: GTGCCACCAGTCCCAGAC

WISP1-5′: GGGATAGCAAGCATCCAGAG;

WISP1-3′: CCTTCATGACACGTGAAAGC

### DNA microarray data preprocessing

All the array datasets were downloaded from public domains, and were MIAME compliant. Mouse and MEF expression data was available through NCBI's Gene Expression Omnibus (GEO) with accession IDs GSE29010 and GSE1413, and normalized by RMA method. Human expression data sets were downloaded from GEO and other public domains (see Text S1). If a gene has multiple probesets representing it, its expression was evaluated as the average of its probesets.

### Transcription factor activity (TFA) analysis

NCA is built based on log-linear model in which gene expression ratios are log-linearly proportional to activity ratios of their regulators. In the NCA pre-processing steps, expression data sets from single channel Affymetrix arrays were set in log ratios comparing the conditions of interest (e.g. Pten null, hi-Myc, tumors) to the references (e.g. WT, normal). Biological repeat arrays were averaged first before calculating the log ratios to filter out extreme value of log-ratio.

We used the network structure information provided by Transcriptional Regulatory Element Database (TRED) from Cold Spring Harbor Laboratory [55], in which the connectivity information is based on experimental validation and motif searching. NCA and trimming algorithm [16] were used to reconstruct the transcriptional network for each data set. In the study, we only used the experimental-based TF-gene relationships to minimize the false positive connection defined by motif searching. The statistical significance of TFAs in murine datasets was justified based on random sampling network expression [16]. In brief, we first generate n∼200 random networks whose gene expression data is random sampled from whole genome. Such random networks are then analyzed by NCA using the same network structure as the interested one. The collection of TFAs obtained from analyzing random networks form the null distributions. For each original TFA in individual experiments, the TFA modified z-score was calculated based on the median and median of absolute deviation to determine how significantly the TFA is perturbed under the experimental conditions. The median and median of absolute deviation, instead of mean and standard deviation, are used to avoid the effect of outliners.

### Clustering analysis

Cluster 3.0 was used for the unsupervised hierarchical clustering analysis using TFAs/gene expression. The similarity between samples was represented by the cosine (or un-centered correlation) metric. Complete linkage was used to clusters samples. In the heatmap of prostate data the TFAs are colored code based on their relative values to the respective averages of normal samples to illustrate the direction of TFA variation to normal prostate tissue.

### Pair wise correlation coefficient analysis

In each human cancer dataset, Pearson correlation coefficient between each pair of TFAs was calculated. The unsupervised hierarchical clustering analysis was then used to rearrange the order of TFAs in the matrix of the absolute correlation coefficients.

### Western blot analysis

Whole-cell extraction was described detail in [21]. Cell lysates were subjected to 10% sodium dodecyl sulfate-polyacrylamide gel electrophoresis, transferred onto nitrocellulose (Bio-Rad), and followed by Western blot analysis using PTEN (9552; Cell Signaling), c-MYC (Santa Cruz), STAT6 (Santa Cruz), p-STAT6 (Abcam), JUN and p-JUN (Cell Signaling) antibodies. Quantification was performed with BioRad Image Lab software.

## Supporting Information

Figure S1 **Validation of PTEN-inducible systems.** (A) Restoration of *PTEN* expression suppressing the expression of the p90 isoform, but not p76 isoform of MDM2 in *PTEN* inducible PC3 cells. (B) *PTEN* re-expression does not change the c-MYC, STAT6 and c-JUN total protein levels but does alter the ratio of phosphor-c-JUN to total c-JUN. Numbers indicate the relative ratio of phosphorylated to total protein, or the levels of c-Myc protein, with the PTEN null state defined as unity.
(TIFF)

Figure S2 **Heatmap of TFAs changes deduced from gene expression profiles in PTEN inducible MEFs and prostate cancer mouse models.** Heatmap showing PTEN-controlled TFAs that are significantly altered in PTEN-inducible MEF tissue culture cells; and a set of prostate cancer-related TFAs that are significantly altered during tumorigenesis in murine prostate cancer models, but not by re-expression of PTEN in the PTEN-inducible MEF system (Rapa: Rapamycin treatment).
(TIF)

Figure S3 **The PTEN-control TFA-based unsupervised clustering analysis.** Unsupervised clustering analysis, based on PTEN-controlled TFAs, was used to classify human tumor samples. (A) the first and (B) the second (NKI) breast cancer data sets and in (C) brain cancer dataset. In the first breast tumor data set (A), PTEN-controlled TFA-based unsupervised clustering yields a clustering pattern of tumor PTEN negative status (Group 1). As for the second breast cancer data set the dendrogram also illustrates the association of PTEN-negative Group 1 with poorly differentiated, ER-negative and basal-like phenotype.
(TIF)

Figure S4 **NCA-inferred TFAs significantly altered in human breast cancer based on PTEN IHC.** Log_10_-transformed t-test p values for each TFA between samples of different IHC-based PTEN status. The graph shows the 45 TFs with the highest log_10_-transformed p-values. The p-values >0.1 of the other 25 TFs are not shown. The dashed line (p = 1e-4) indicates the threshold value for selecting the TFA-based PTEN-IHC-derived signatures used in the analysis in Figure 5 (gold).
(TIF)

Table S1 **List of PTEN immediately controlled genes in MEFs.**
(XLS)

Text S1 **Supporting Information.**
(DOC)
