# Supplementary figures and images for: Determining PTEN Functional Status by Network Component Deduced Transcription Factor Activities
Source: PLoS One. 2012 Feb 8;7(2):e31053. doi: 10.1371/journal.pone.0031053 (PMC3275574; doi:10.1371/journal.pone.0031053)

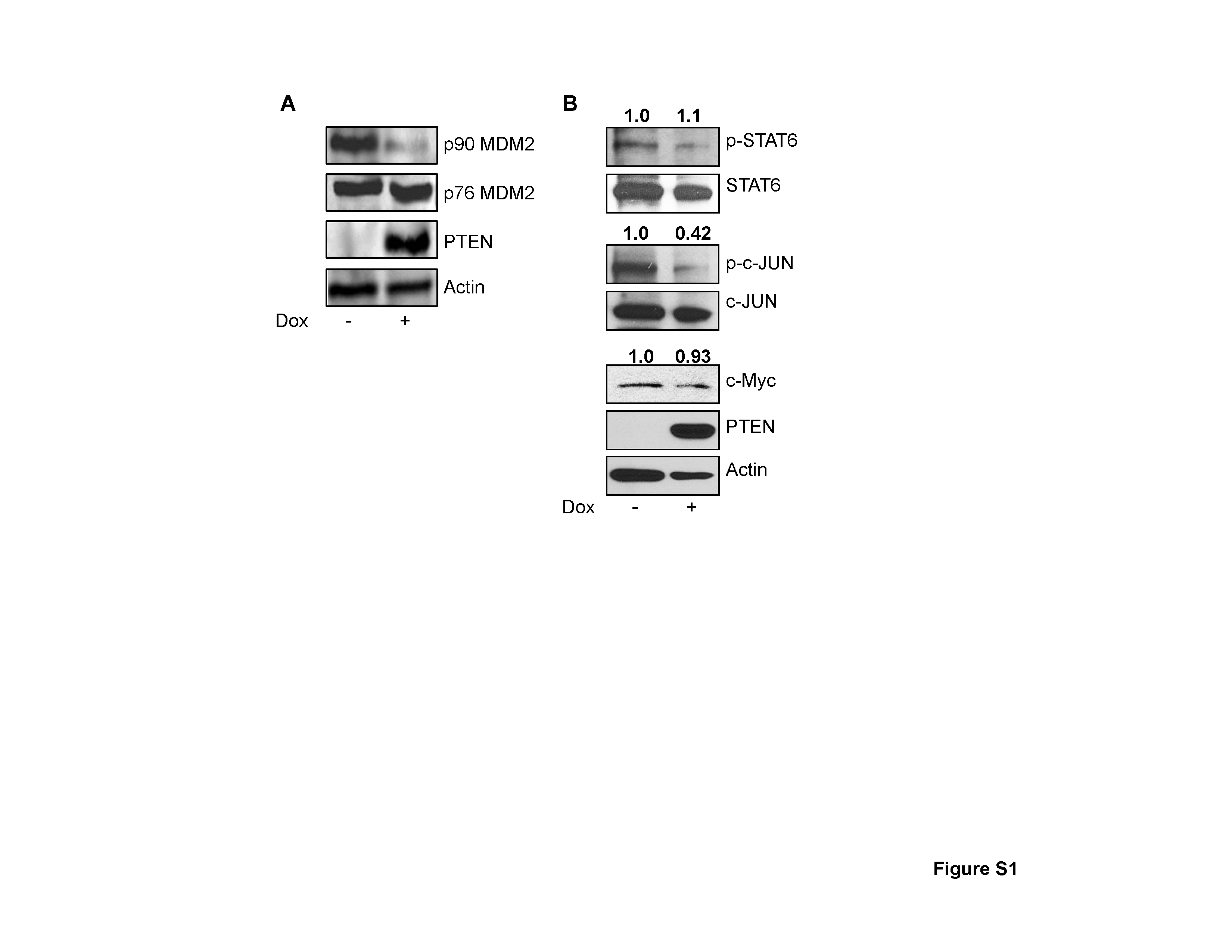

Supplement: Figure S1 — Validation of PTEN-inducible systems. (A) Restoration of PTEN expression suppressing the expression of the p90 isoform, but not p76 isoform of MDM2 in PTEN inducible PC3 cells. (B) PTEN re-expression does not change the c-MYC, STAT6 and c-JUN total protein levels but does alter the ratio of phosphor-c-JUN to total c-JUN. Numbers indicate the relative ratio of phosphorylated to total protein, or the levels of c-Myc protein, with the PTEN null state defined as unity. (TIFF) [file pone.0031053.s001.tiff]

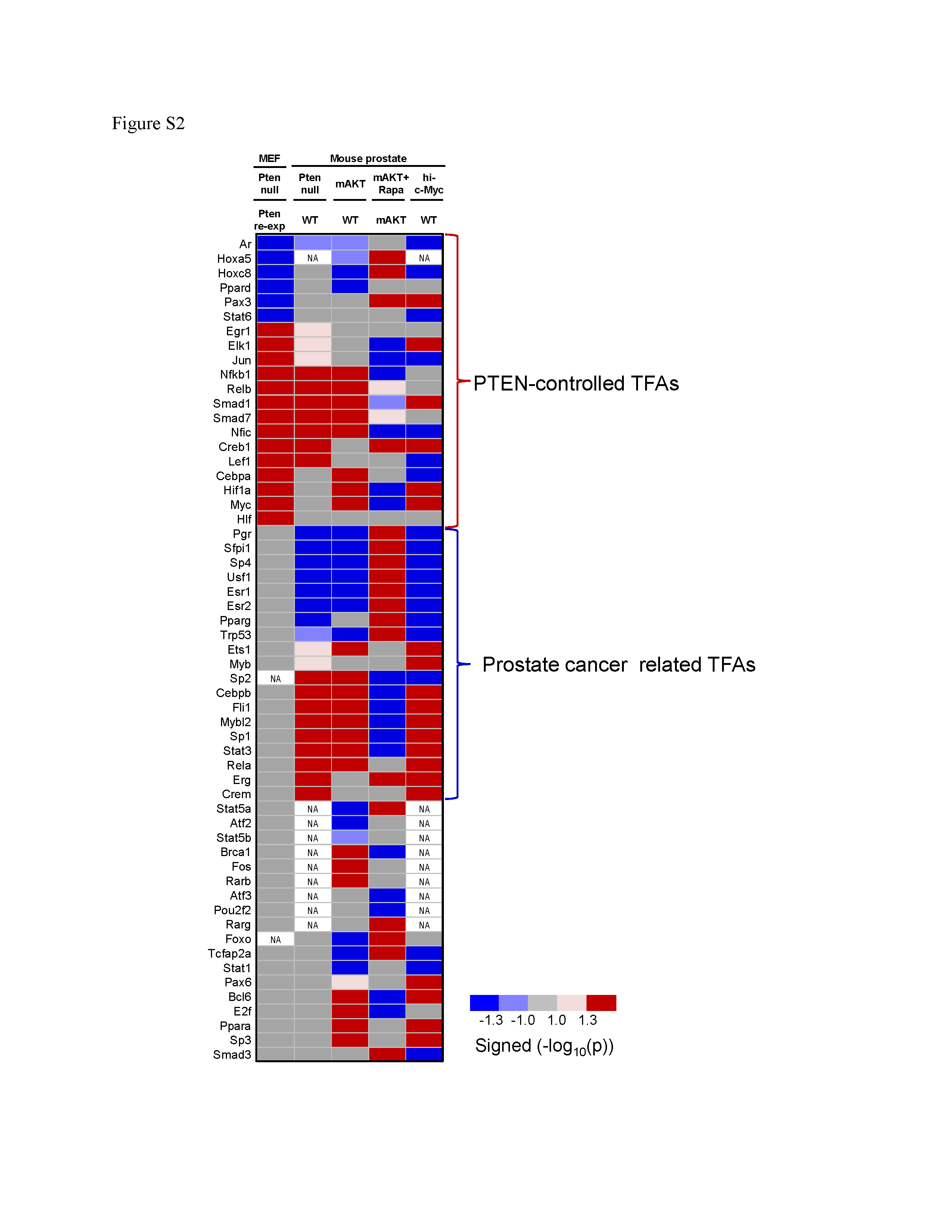

Supplement: Figure S2 — Heatmap of TFAs changes deduced from gene expression profiles in PTEN inducible MEFs and prostate cancer mouse models. Heatmap showing PTEN-controlled TFAs that are significantly altered in PTEN-inducible MEF tissue culture cells; and a set of prostate cancer-related TFAs that are significantly altered during tumorigenesis in murine prostate cancer models, but not by re-expression of PTEN in the PTEN-inducible MEF system (Rapa: Rapamycin treatment). (TIF) [file pone.0031053.s002.tif]

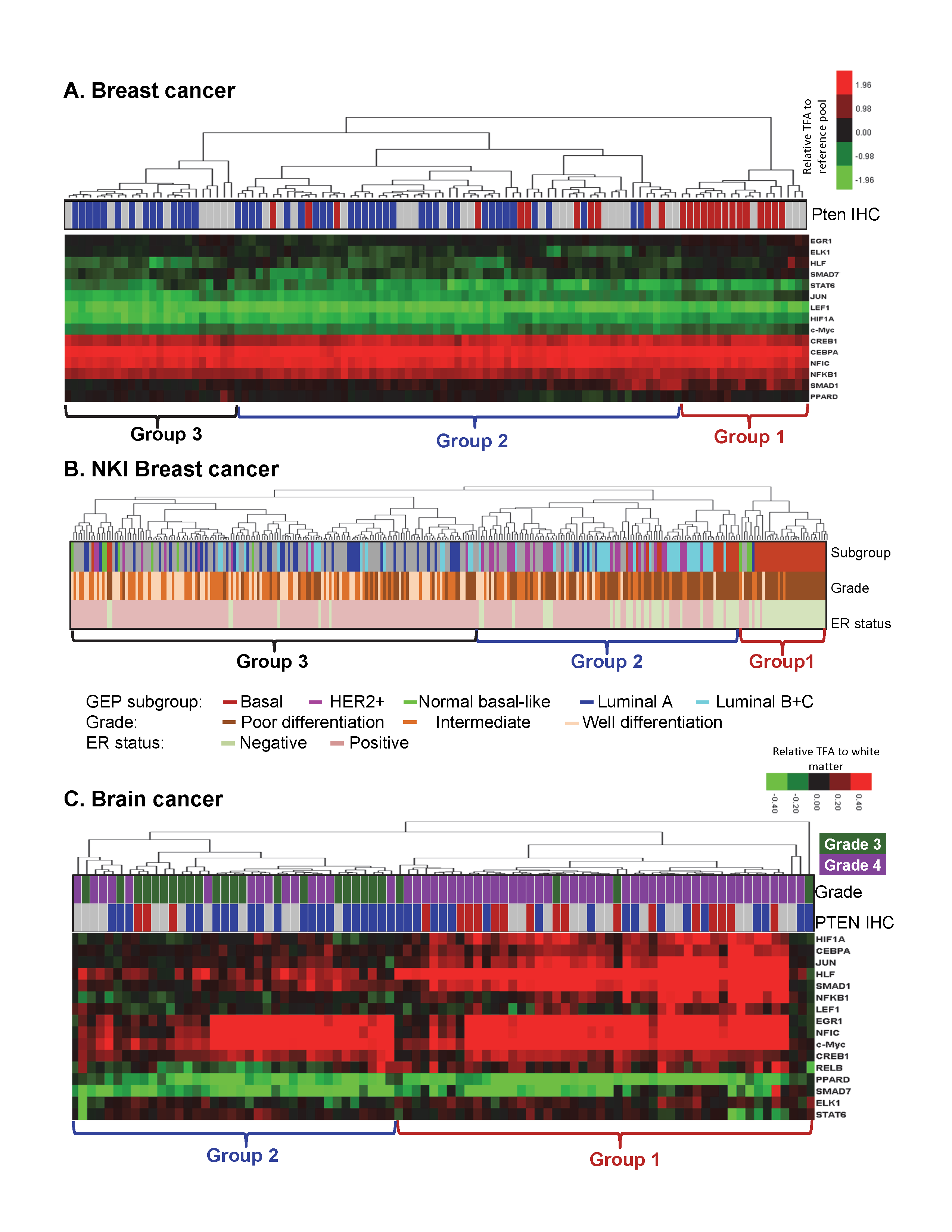

Supplement: Figure S3 — The PTEN-control TFA-based unsupervised clustering analysis. Unsupervised clustering analysis, based on PTEN-controlled TFAs, was used to classify human tumor samples. (A) the first and (B) the second (NKI) breast cancer data sets and in (C) brain cancer dataset. In the first breast tumor data set (A), PTEN-controlled TFA-based unsupervised clustering yields a clustering pattern of tumor PTEN negative status (Group 1). As for the second breast cancer data set the dendrogram also illustrates the association of PTEN-negative Group 1 with poorly differentiated, ER-negative and basal-like phenotype. (TIF) [file pone.0031053.s003.tif]

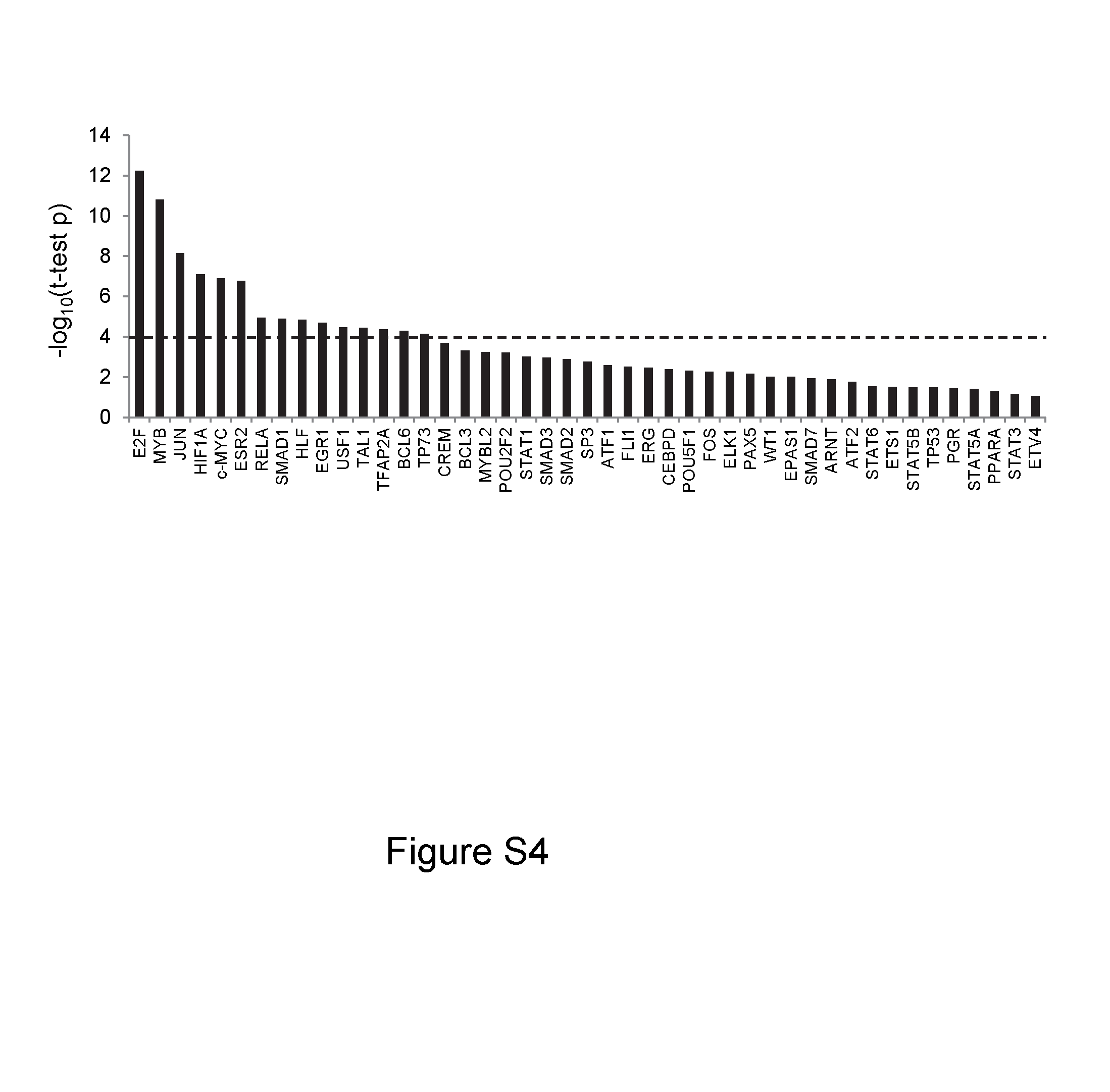

Supplement: Figure S4 — NCA-inferred TFAs significantly altered in human breast cancer based on PTEN IHC. Log10-transformed t-test p values for each TFA between samples of different IHC-based PTEN status. The graph shows the 45 TFs with the highest log10-transformed p-values. The p-values >0.1 of the other 25 TFs are not shown. The dashed line (p = 1e-4) indicates the threshold value for selecting the TFA-based PTEN-IHC-derived signatures used in the analysis in Figure 5 (gold). (TIF) [file pone.0031053.s004.tif]
