# Supplementary material for: Determining PTEN Functional Status by Network Component Deduced Transcription Factor Activities
Source: PLoS One. 2012 Feb 8;7(2):e31053. doi: 10.1371/journal.pone.0031053 (PMC3275574; doi:10.1371/journal.pone.0031053)
Supplement: Text S1 — Supporting Information. (DOC) [file pone.0031053.s006.doc]

**Supporting Information**

**Processing of microarrays.**

Mouse and MEF expression datasets were available through Gene Expression Omnibus (GEO) with accession IDs GSE29010 and GSE1413 respectively. The human data sets were collected from different public domains. The human breast cancer was downloaded from GEO accession ID GSE5235, while the prostate data set was from Stanford Microarray Database (<http://smd.stanford.edu/>), and brain cancer data set and its reference normal using white matter were obtained from UCLA genomic department (<https://secure.genome.ucla.edu/index.php/Publications>). For the data sets using Affymetrix platforms, the expression data was normalized by RMA. If a gene has multiple probesets representing it, the average of its probesets was used to representing the gene expression.

**Network Component Analysis**

NCA and trimming algorithm were used to reconstruct the transcriptional network for each data set. In the NCA pre-processing steps, expression data sets from single channel Affymetrix arrays were set in log_2_ ratios comparing the conditions of interest (e.g. Pten null, hi-Myc, tumors) to the references (e.g. WT, normal). The TF-gene information were obtained from Transcriptional Regulatory Element Database (TRED) of Cold Spring Harbor Laboratory , in which the connectivity information is based on experimental validation and motif searching. However, in this study we used the TF-gene relationship derived from experimental validation. For each human dataset, a transcriptome network was reconstructed based on around ~1600 to 2200 genes regulated by 70-80 TFs. In the mouse data sets, the networks have average size of 800 genes regulated by 70 TFs.

**References**

1. Saal LH, Johansson P, Holm K, Gruvberger-Saal SK, She QB, et al. (2007) Poor prognosis in carcinoma is associated with a gene expression signature of aberrant PTEN tumor suppressor pathway activity. Proc Natl Acad Sci U S A 104: 7564-7569.

2. Lapointe J, Li C, Higgins JP, van de Rijn M, Bair E, et al. (2004) Gene expression profiling identifies clinically relevant subtypes of prostate cancer. Proc Natl Acad Sci U S A 101: 811-816.

3. Freije WA, Castro-Vargas FE, Fang Z, Horvath S, Cloughesy T, et al. (2004) Gene expression profiling of gliomas strongly predicts survival. Cancer Res 64: 6503-6510.

4. Tran LM, Hyduke DR, Liao JC (2010) Trimming of mammalian transcriptional networks using network component analysis. BMC Bioinformatics 11: 511.

5. Zhao F, Xuan Z, Liu L, Zhang MQ (2005) TRED: a Transcriptional Regulatory Element Database and a platform for in silico gene regulation studies. Nucleic Acids Res 33: D103-107.
